# Editorial: Brain reward and aversion circuits: progress review on current and future breakthroughs

**DOI:** 10.3389/fnbeh.2025.1632916

**Published:** 2025-06-17

**Authors:** Dan P. Covey, Keith B. J. Franklin

**Affiliations:** ^1^Lovelace Biomedical Research Institute, Albuquerque, NM, United States; ^2^Department of Psychology, McGill University, Montreal, QC, Canada

**Keywords:** reward, aversion, nucleus accumbens, dopamine, circuits

This Research Topic covers a collection of review articles and new experimental results that enhance our understanding of how dopaminergic and other inputs to the nucleus accumbens (NAc) and prefrontal cortex mediate both reward and aversion, and how these systems interact to control behavior. The research addresses key questions about the mechanisms underlying reward and aversion processing in the brain. By examining these interactions, the research seeks to clarify the complex dynamics of reward and aversion circuits and their implications for behavior and potential therapeutic interventions.

Xu et al. provide a comprehensive review of NAc circuitry. Historically seen as a primary site for reward processing, the NAc has less frequently been implicated in aversion processing, as well. The authors describe the anatomical and functional heterogeneity of the NAc, including the various cell types and their interconnections. Xu et al. also discuss how dysregulation within the NAc circuitry can lead to psychiatric disorders such as addiction and depression. Prolonged exposure to stress or drug use can induce maladaptive plasticity, altering synaptic strength and neurotransmitter balance in a way that reinforces compulsive behavior or persistent negative mood. The review calls for a more refined understanding of the pathways and cell types involved, especially the role of neuromodulators like dopamine, serotonin, and acetylcholine, in order to develop targeted treatments that can restore healthy motivational states.

Robison et al. evaluated how intermittent swim stress affects the economic demand for ethanol. They found that all rats, whether they experienced real stress or a sham condition, exhibited increased demand for alcohol following the stress procedure. This suggested that factors such as time-dependent craving or abstinence effects may drive general increases in ethanol consumption. However, when the researchers accounted for physiological stress responses by measuring corticosterone levels, they uncovered a stronger narrative. Rats that showed greater increases in corticosterone following the swim stress also demonstrated greater increases in ethanol demand, revealing a clear correlation between stress responsivity and substance-seeking behavior.

Braden and Castro provide an in-depth overview of the dorsal raphe nucleus (DRN), a region in the midbrain traditionally known for its role as the primary source of serotonin to the forebrain. Yet, the DRN contains a much richer neurochemical environment than previously acknowledged, with a significant proportion of its neurons being non-serotonergic and expressing a variety of neuropeptides. Within the DRN, peptides such as dynorphin, enkephalin, nociception, corticotropin-releasing factor, galanin, and neuropeptide Y contribute to modulating reward, stress, and aversion. The authors detail how these molecules influence both local circuits within the DRN and projections to other regions like the amygdala, NAc, and ventral tegmental area. Notably, these peptide systems often operate independently of serotonin, challenging the monoamine-centric view of mood regulation that has dominated psychiatric medicine for decades.

Deng et al. provide a sweeping review of the neurobiological mechanisms underpinning reward prediction error (RPE) and its significance in learning and adaptation. They explain that learning is essentially a process of updating beliefs in response to surprises, and that dopamine neurons, particularly in the ventral tegmental area and substantia nigra pars compacta, are instrumental in signaling these surprises. Deng et al. delve into how this neural mechanism supports various forms of learning, such as reinforcement learning and reversal learning. The review also connects RPE signaling to disorders such as Parkinson's disease, where dopamine deficits impair this fundamental learning process, and addiction, where drugs hijack the reward system, creating exaggerated RPEs that reinforce harmful behaviors. Furthermore, the authors highlight that RPE not only aids in the acquisition of new behaviors but also in memory reconsolidation, where old memories are updated when mismatched information is encountered. Through this lens, RPE is not just a learning signal, but also as a tool for cognitive flexibility, enabling organisms to revise their expectations in a dynamic world.

Ihara et al. address a common challenge in studying motivation: how to interpret complex choice behavior in animals beyond a single data point. In progressive ratio (PR) tasks, the effort required to obtain a reward increases over time, and motivation is typically measured by the “breakpoint,” or the maximal effort an animal will pay for a reward. However, Ihara et al. argue that this measure oversimplifies the complex stream of decisions animals make during the entire task. To better capture this nuance, they developed several computational models and tested them using behavioral data from mice engaged in PR tasks. The most accurate model was one that included “choice traces” or a memory of recent actions that influences future decisions. This model predicted that magazine nosepokes (where a mouse checks for a reward before completing the required effort) should diminish over time if they are not reinforced, a prediction validated by real-time dopamine measurements in the ventral striatum using fiber photometry. Interestingly, when mice were administered a low dose of methamphetamine the frequency of these nosepokes increased, though the breakpoint remained unchanged. This subtle behavioral modulation, captured through their model, underscores its potential to detect effects that conventional analysis might miss.

In shifting from computational neuroscience to behavioral intervention, the final article by Shen et al., offers a broader, translational perspective. Their systematic review and meta-analysis examine whether voluntary wheel-running can alleviate depressive symptoms in rodents. The researchers reviewed data from 15 studies, covering multiple models of depression, including maternal separation, chronic unpredictable mild stress, restraint stress, and social defeat. Across these varied paradigms, wheel-running consistently improved performance on classic behavioral assays for depression and anxiety. While the benefits of exercise were clear, the authors also noted substantial heterogeneity in study design, such as differences in exercise duration, stress induction protocols, and behavioral assessments. Nevertheless, the results converged on the same conclusion: voluntary physical activity has a robust and beneficial effect on mood-related behaviors in rodents. The mechanisms are likely multifactorial—ranging from enhanced dopamine and serotonin transmission to increased neuroplasticity and anti-inflammatory effects—many of which overlap with the same neural pathways involved in RPE and motivation.

